# Awakening the dormant: Role of axonal guidance cues in stress-induced reorganization of the adult prefrontal cortex leading to depression-like behavior

**DOI:** 10.3389/fncir.2023.1113023

**Published:** 2023-03-24

**Authors:** Ashraf Mahmud, Radu Gabriel Avramescu, Zhipeng Niu, Cecilia Flores

**Affiliations:** ^1^Integrated Program in Neuroscience, McGill University, Montréal, QC, Canada; ^2^Douglas Mental Health University Institute, Montréal, QC, Canada; ^3^Department of Psychiatry, Neurology, and Neurosurgery, McGill University, Montréal, QC, Canada

**Keywords:** Netrin-1, social defeat, post-mortem, MDD, ephrin, slit, DCC, semaphorin

## Abstract

Major depressive disorder (MDD) is a chronic and disabling disorder affecting roughly 280 million people worldwide. While multiple brain areas have been implicated, dysfunction of prefrontal cortex (PFC) circuitry has been consistently documented in MDD, as well as in animal models for stress-induced depression-like behavioral states. During brain development, axonal guidance cues organize neuronal wiring by directing axonal pathfinding and arborization, dendritic growth, and synapse formation. Guidance cue systems continue to be expressed in the adult brain and are emerging as important mediators of synaptic plasticity and fine-tuning of mature neural networks. Dysregulation or interference of guidance cues has been linked to depression-like behavioral abnormalities in rodents and MDD in humans. In this review, we focus on the emerging role of guidance cues in stress-induced changes in adult prefrontal cortex circuitry and in precipitating depression-like behaviors. We discuss how modulating axonal guidance cue systems could be a novel approach for precision medicine and the treatment of depression.

## Introduction

Major depressive disorder (MDD), a multifactorial disorder with heterogeneous symptomatology, is one of the leading causes of disease and disability worldwide, affecting roughly 280 million people, and manifesting an estimated lifetime prevalence of approximately 17% (Kessler et al., 2003; James et al., 2018; Johnston et al., 2019; Gold et al., 2020; World Health Organization, 2021). Despite decades of research, about 50% of depressed patients are not adequately covered by existing interventions (Akil et al., 2018), and about 30–50% of the patients who receive treatment eventually relapse (de Zwart et al., 2018). Decades of clinical and preclinical studies consistently report that depression is a circuit-level disorder, often induced by chronic stress (Pizzagalli, 2014; Heshmati and Russo, 2015; Bagot et al., 2016; Fischer et al., 2016; Belleau et al., 2018; Hare and Duman, 2020; Spellman and Liston, 2020). Chronic stress disrupts the organization of the prefrontal cortex (PFC) and its connections with cortical, subcortical, and limbic areas–a phenomenon associated with increased susceptibility to depression in humans and to depression-like behavioral abnormalities in rodents (Liston et al., 2009; McEwen and Morrison, 2013; Liu et al., 2017; Hare and Duman, 2020). At the cellular level, chronic stress induces circuitry alterations by impairing signaling essential for the maintenance and formation of neuronal connections, as well as synaptic transmission and plasticity (Duman and Aghajanian, 2012; Chaudhury et al., 2015; Duman et al., 2016; Yan and Rein, 2022). Guidance cues known to be involved in the organization and plasticity of synaptic networks are pre-eminently important in these cellular processes and may act as molecular links between stress and PFC circuitry remodeling (Dickson, 2002; Lanoue and Cooper, 2019).

Axonal guidance cues, and their corresponding receptors, are highly conserved families of proteins that steer growing neurites (e.g., axons, dendrites) to their intended targets, dictating the fine organization of neuronal circuits (Dickson, 2002; Guan and Rao, 2003; Stoeckli, 2018; Lanoue and Cooper, 2019). These cues include classical guidance molecules such as the netrin, ephrin, slit, and semaphorin families, and non-conventional molecules such as morphogens including sonic hedgehog (*SHH*), bone morphogenetic proteins (*BMP*s), and the wingless-type family (*WNT*s). In general, however, not exclusively, axonal guidance cues are secreted, diffusible proteins that are highly expressed during early brain development, both in embryonic and early post-natal life, when they guide long-distance axonal pathfinding required for proper brain wiring and maturation (Dickson, 2002; Guan and Rao, 2003; Stoeckli, 2018). Secreted guidance cues can also bind to the extracellular matrix and function as local haptotactic adhesive signals through one of two main mechanisms: affinity to a component of the extracellular matrix (e.g., Netrin-1 binds to heparan sulfate proteoglycans) (Kennedy et al., 1994; Matsumoto et al., 2007); and surface adhesion to a membrane bound molecule (e.g., Slit-2 adsorbed to GPI-anchored heparan sulfate proteoglycan glypican-1) (Lau and Margolis, 2010; Dominici et al., 2017; Varadarajan et al., 2017; Boyer and Gupton, 2018; Meijers et al., 2019). Expression of axonal guidance cues is at its maximum during embryonic and fetal development, when the forming brain is involved in intensive long-distance wiring. This high expression decreases in post-natal life, with additional evidence from mice of changes between adolescence and adulthood in a number of guidance cue pathways (netrin, ephrin, semaphorin) (Goldman et al., 2013), as the matured brain attenuates large-scale rewiring, but they continue to play a critical role in the organization of local circuits by influencing dendritic structure and synaptic plasticity (Stoeckli, 2018; Lanoue and Cooper, 2019; Glasgow et al., 2020; Torres-Berrío et al., 2020a). Many guidance cues diffuse out from their site of production, often establishing a concentration gradient, and interacting with their specific receptors on the growth cone of the growing neurite. Interaction with specific receptors at this site can induce attraction and neurite growth or can trigger repulsion and neurite collapse, depending on the particular ligand: receptor pair (Dent et al., 2011; McCormick and Gupton, 2020).

Recent studies in rodents have demonstrated that exposure to chronic stress leads to dysregulation of axonal guidance cue expression, which leads to depression-like behavioral states (Torres-Berrío et al., 2020a; Vosberg et al., 2020; Van der Zee et al., 2022). We propose that chronic stress-induced changes in axonal guidance cue function trigger the reorganization of local PFC circuitry increasing incidence of behavioral abnormalities in rodents and the onset and severity of depression in humans. In this review, we discuss axonal guidance cue systems as important mediators of persistent effects of stress exposure in adulthood on PFC neuronal connectivity and behavior, with special consideration given to sex-dependent divergent results, to the extent to which they have been investigated. Within this framework, our focus will mainly be the classical guidance cue families, Netrins, Slits, Ephrins, and Semaphorins, with particular consideration given to Netrin-1, arguably the most studied guidance cue, as well as touching briefly on unconventional guidance cues (morphogens, adhesion proteins). We gather emerging evidence from rodent models for adult chronic stress and from studies in humans indicating altered expression of guidance cues in rodents susceptible to stress and in individuals with MDD, when pertinent, touching on sex differences and their possible protective or predisposing features.

## Stress exposure in adulthood reduces PFC synaptic connections in rodents

The PFC is significantly larger in humans, compared to rodents, due to the developmental expansion of the dorsolateral PFC and the frontal pole, seen in primates, but absent in rodents (Wise, 2008). The PFC in rodents consists of medial, orbitofrontal and cingulate areas and lacks an anatomical homolog of the primate dorsolateral PFC (Kolb et al., 2012; Kolk and Rakic, 2022). Nonetheless, the rodent prelimbic and infralimbic subregions of the PFC are considered functionally homologous to the human pregenual anterior cingulate cortex (Brodmann area 24) and the subgenual cingulate cortex (Brodmann area 25), both widely shown to be involved in MDD (Uylings et al., 2003; Kolb et al., 2012; Pizzagalli and Roberts, 2022). The PFC is highly implicated in reward, motivated behavior, memory, decision-making, and reinforcement learning (Fuster, 2002; Alexander and Brown, 2011; Euston et al., 2012; Wang et al., 2018) while also required for top-down processing, cognitive control, the internal representation of goals, goal-directed behavior, and planning. Dysfunction of the PFC in humans leads to impulsive, disorganized, and socially inappropriate behavior (Wise et al., 1996; Miller, 1999; Miller and Cohen, 2001; Dalley et al., 2004). As part of the frontal lobes, the PFC is one of the last brain areas to fully mature, with its development completing well into adulthood (Sowell et al., 1999; Fuster, 2002; Gogtay et al., 2004; Parker et al., 2020). It is plausible that, due in part to its protracted critical maturation period, the PFC is uniquely vulnerable to stress-induced insults.

The dysfunction of the PFC is well documented in MDD and in rodent models for chronic stress-induced depression-like behavioral abnormalities, including the adult chronic social defeat paradigm (CSDS) (George et al., 1994; Czéh et al., 2007; Ma et al., 2016; Murrough et al., 2016; Liu et al., 2017; Belleau et al., 2018). CSDS induces alterations in the function of the PFC circuit, which augment the susceptibility to developing social and motivational deficits (Vialou et al., 2014; Bagot et al., 2015; Bonnefil et al., 2019). More precisely, exposure to chronic stress induces loss of dendrites and spines in PFC neurons, and these changes are associated with impairments in PFC functioning and PFC-mediated behaviors such as decision-making and attentional set-shifting (Radley et al., 2005, 2006; Liston et al., 2006; Dias-Ferreira et al., 2009; Hains et al., 2009; Shansky et al., 2009). These structural modifications do not appear to be permanent, since after long periods in the absence of stress, the number of dendrites and spines increases back to baseline levels (Radley et al., 2005). The neuronal changes mentioned are not pleiotropic throughout the PFC, but are circuit specific. For example, Shansky et al. (2009) found that chronic stress induces a reduction in dendritic number in PFC neurons that have cortico-cortical projections but leads to an increase in the number of dendrites of PFC neurons innervating the amygdala. The concentration of circulating blood estrogen as dictated by biological sex has also emerged as an important factor. The same group showed, using a model for pharmacological activation of the stress system, that female rats are more sensitive than males to developing PFC-dependent working memory deficits, but only during the period of high levels of circulating estrogen. Ovariectomized females had lower stress sensitivity, but regained high sensitivity to stress after exogenous estrogen replacement (Shansky et al., 2004, 2010). Sexually dimorphic changes in adult PFC synaptic connectivity induced by chronic stress which are dependent on circulating gonadal hormones have also been shown using various paradigms. Many types of chronic stress ultimately result in removal of PFC synapses, suggesting that different stressors produce a similar outcome, possibly through a shared mechanism (Popoli et al., 2012; Wellman and Moench, 2019; Woo et al., 2021). While the endpoint effector pathway has not yet been identified for the PFC, in the hippocampus, the expression of the cell adhesion protein PSA-NCAM has been proven to decrease in rodent models of depression-like behaviors, while the effectiveness of antidepressants correlates with increased PSA-NCAM in the hippocampus (Wainwright and Galea, 2013). We argue that understanding the molecular players involved in stress-induced synaptic connectivity changes in the PFC is fundamental for developing novel therapeutic approaches.

## Models for chronic stress-induced depression-like behaviors in adult rodents help elucidate the molecular pathology of MDD in humans

**FIGURE 1:**
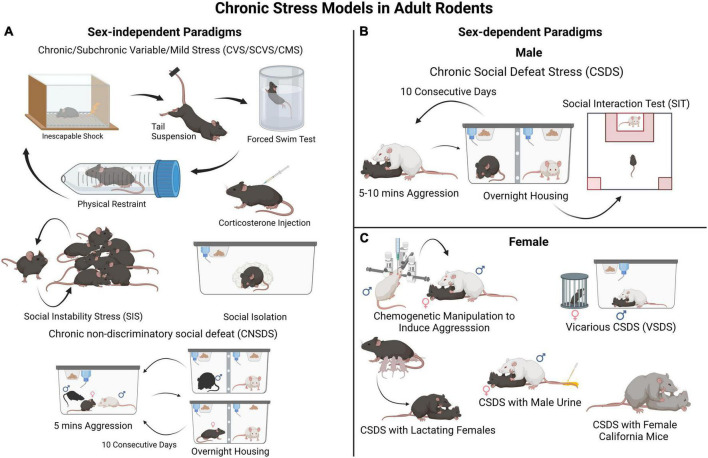
Rodent models for chronic stress-induced depression-like behaviors. **(A)** These are examples of commonly employed sex non-specific paradigms such as different variations of chronic/subchronic variable (mild) stress (chronically exposing the experimental animals to different stressors in random order), social instability stress (disturbances in an animal’s social hierarchy), social isolation, and corticosterone administration. These can be used in both male and female mice to investigate biological sex-mediated mechanisms in stress vulnerability. In these paradigms animals are not segregated into resilient and susceptible groups, thus examination of individual differences in stress susceptibility is limited. **(B)** A widely used and validated model of chronic stress in male mice is CSDS. In this procedure, control animals are housed with a conspecific and a divider that allows sensory stimuli. Each experimental male mouse is exposed to a social defeat session (for 5–10 min each day) in which the mouse is socially defeated by an aggressive CD-1 mouse for several (often 10) consecutive days. After the CSDS, all mice are assessed in a social interaction test to evaluate their sociability toward a social target (e.g., a novel CD-1 mouse). This will classify each experimental mouse in either susceptible or resilient phenotype. **(C)** A major limitation of the CSDS model is that female mice are not readily attacked by an aggressor CD-1 mouse. Thus, the CSDS model has needed to be adapted for female mice. A few different approaches have been described, such as a modified version of CSDS involving Swiss Webster female mice (Newman et al., 2019; Williams et al., 2022) and CSDS with lactating females, (Bourke and Neigh, 2012; Jacobson-Pick et al., 2013) both shown to induce behavioral deficits in female mice. However, the defeated mice are not segregated into resilient and susceptible groups, which limits in-depth assessment. An additional paradigm involves marking female experimental mice with male urine to induce aggression from CD-1 mice within a CSDS framework (Harris et al., 2018). Therefore, it can be used in parallel with classical CSDS in males to investigate social stress vulnerability in both sexes and identify divergent effects either at the behavioral or molecular levels.

Much of our understanding of the cellular and molecular processes involved in the development of depression-like behavior comes from studies using rodents. Modeling chronic stress entails exposing rodents to prolonged stress, such as social stress, physical restraint or inescapable foot shock, direct activation of the stress system, or to various types of stressors (wet bedding, loud noise) presented in a variable sequence to prevent habituation effects (Newman et al., 2018). The effect of stress on anhedonia, learned helplessness, social avoidance, appetite and body weight, sleep and circadian rhythms, self-care/grooming, among several other measures, are assessed to determine the impact of stress on depression-like behavioral states (Nestler and Hyman, 2010; Krishnan and Nestler, 2011; Czéh et al., 2016; Wang et al., 2017; Antoniuk et al., 2018; Torres-Berrío et al., 2020a). It should be noted that some of the behavioral and physiological alterations induced in these rodent paradigms reproduce aspects of the traits observed in MDD, helping us understand how brain function and behavior change in response to chronic stress. However, it should be emphasized that these rodent models mimic neither depressive symptoms, as experienced by humans, nor the complex interaction between genetic and environmental factors. Often, following chronic stress exposure, susceptible and resilient phenotypes are assessed in behavioral tests that measure phenotypic traits such as reward-seeking and reward sensitivity, learning, social preference and/or interaction, reward preference, and time spent grooming (Nestler and Hyman, 2010; Krishnan and Nestler, 2011; Czéh et al., 2016; Wang et al., 2017; Antoniuk et al., 2018; Torres-Berrío et al., 2020a). Figure 1 depicts some of the most commonly employed models for chronic stress used in adult male and female mice. Despite the limitations of using rodents, results from stress models have revealed changes in neural circuitry in brain areas implicated in MDD to be associated with resilience/susceptibility to stress (Frazer and Morilak, 2005; Nestler and Hyman, 2010; Wang et al., 2017; Bale et al., 2019; Parekh et al., 2022).

While several models for chronic stress can be implemented in either male or female rodents (Figure 1A), some of them have only been applied to males, particularly those developed for mice (Figure 1B). One of these models is the CSDS paradigm in which an adult C57BL/6 experimental male mouse is subjected to repeated physical attacks and submission by an aggressive CD-1 conspecific male. Defeated mice can then be classified as “susceptible” or “resilient” based on their social approach phenotype in a social interaction test performed typically 24 h after the last defeat session (Figure 1B). Susceptible mice also show deficits in other behavioral domains linked to MDD, for example anhedonia (Berton et al., 2006; Krishnan et al., 2007; Golden et al., 2011). A limitation of the standard CSDS model is that adult females are not attacked by an aggressive conspecific, which hinders implementation in females of models designed for male mice. Due to this difference between male-male and male-female aggression, it has been difficult to run social defeat protocols identically for both male and female mice, a known confounding factor in the field. However, to overcome this limitation, new models adapted for females are emerging, including chemogenetically activating the ventrolateral subdivision of the ventromedial hypothalamus of the CD-1 aggressor to induce attacks toward females (Takahashi et al., 2017), applying urine from male mice to experimental females to induce aggression from CD-1 mice, (Harris et al., 2018) vicarious social defeat stress model (Iñiguez et al., 2018), and the chronic non-discriminatory social defeat stress model (Figure 1C; Yohn et al., 2019). Due, in part, to our extensive work with mouse models, we, and others, posit that stress models for females and males do not need to be identical for them to generate meaningful insights into stress pathophysiology, and can be adapted depending on the questions asked (Lopez and Bagot, 2021). For example, standard CSDS in males and chronic variable stress in females can be used to investigate sex-specific molecular mechanisms whereas chronic non-discriminatory social defeat stress can be used both in male and female mice simultaneously to investigate sex-dependent mechanisms and potential sex-differences.

## Role of guidance cues in stress-induced PFC dysfunction and depression

**FIGURE 2:**
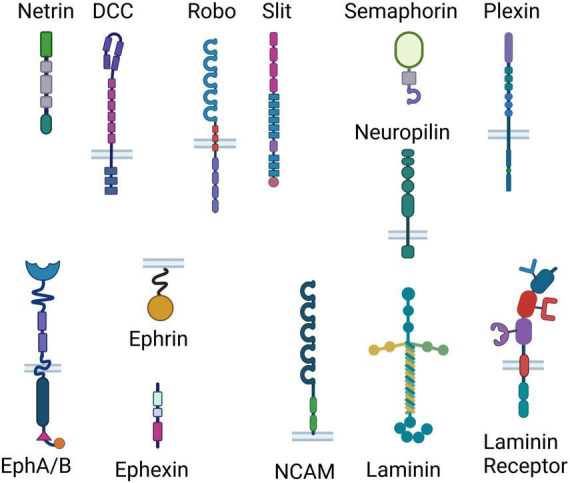
Representation of the classical guidance cue systems and adhesion proteins involved in axonal guidance. Different effects of chronic stress ultimately result in removal of synapses and reduction in neurotransmission, suggesting that different stressors may induce the same outcome through shared mechanisms. Here we show cartoon representations of the domain arrangements of the classical guidance cue families (Netrin, Slit, Semaphorin, Ephrin/Ephexin) and their receptors (inserted in bilayer), as well as cell adhesion proteins (laminin, NCAM and laminin receptor). From top to bottom, Netrin consists of a laminin-like domain, 3 epidermal growth factor (EGF) domains, and a Netrin-like domain; DCC consists of 4 Ig (immunoglobulin) domains, 6 fibronectin III-like (FN) domains, a transmembrane region and 3 P-motifs; Robo consists of 5 Ig domains, 3 FN domains, a membrane spanning region and 4 proline-rich regions; Slit consists of 4 leucine-rich repeats, 6 EGF-like domains, a laminin G-like module, 3 EGF-like domains and a cysteine knot; Semaphorin consists of a Sema domain, a PSI (plexin, semaphorin, and integrin) domain and an Ig domain; Neuropilin consists of 2 Sema binding domains, 2 VEGF (vascular endothelial growth factors) binding subdomains, a MAM (meprin, A-5 protein, and receptor protein-tyrosine phosphatase mu) domain, a transmembrane domain and a PDZ [post synaptic density protein (PSD95), drosophila disc large tumor suppressor (Dlg1), and zonula occludens-1 protein (zo-1)] domain; Plexin consist of a Sema domain, 3 PSI domains, 3 IPT (Ig-like, plexins, transcription factors) domains, a transmembrane region, a GAP (GTPase-accelerating proteins) domain, an RBD (Rho-GTPase binding domain) sequence and a GAP domain; the Ephrin receptor A/B consists of an ephrin binding, and EGF-like domain, two FN domains, a membrane spanning region, a kinase domain, a SAM (Sterile alpha motif) domain and a PDZ-binding domain; Ephrin consists of a membrane spanning region and a receptor binding region; Ephexin consists of a DH (Dbl homology) domain, a PH (Pleckstrin homology) domain and a SH3 (SRC Homology 3) domain; laminin consists of 3 laminin domains in the alpha chain, and 2 laminin domains in both the beta and the gamma chains, a coiled coil and 5 laminin G domains; NCAM consists of 5 IgG-like domains and two FN domains; Laminin receptor consists of single-chain variable fragment (scFv) Ig antibody binding domain, a heparin/laminin binding domain, a laminin/PrP binding domain, a transmembrane domain, and a N-terminal domain.

Axonal guidance cue systems play critical roles in the development and maturation of the PFC and its circuitry, with more recent evidence implicating them in dysfunction in the adult brain (Schubert et al., 2015; Yamagishi et al., 2021; Kolk and Rakic, 2022). Multiple neuronal inputs form very selective synaptic connections with local PFC neurons, essential for proper PFC functioning. Classical guidance molecule families such as the netrin, ephrin, slit, and semaphorin (cartoon representation in Figure 2) orchestrate the formation of PFC functional networks across the lifespan and perform maintenance roles of these connections in later life. Apart from conventional axonal guidance cues, morphogens, growth factors and cell adhesion proteins (Figure 2) are also involved in proper target recognition of PFC efferents and in synapse formation (Schubert et al., 2015). Growth factors and morphogens–fibroblast growth factors (*FGFs*), *BMPs*, *SHH*, and *WNTs*–work in tandem with conventional guidance cue systems and adhesion proteins to pattern and steer PFC formation, including integration of connections of various neurotransmitter systems within the PFC) (Schubert et al., 2015). Disruptions to this coordinated process can lead to altered neuronal connectivity and, in turn, behavior. Therefore, it is known that experiences capable of altering guidance cue function can induce changes in the formation of developing neuronal networks, which can translate into behavioral abnormalities later on in life. To govern neuronal connectivity and plasticity, the adult brain uses the very same axonal guidance cues involved in the proper wiring and pathfinding of developing neurons to control dendritic arborization and synapse formation in adulthood (Glasgow et al., 2020). Since guidance cues continue to be expressed in the adult brain, it is conceivable that exposure to chronic stress alters guidance cue function to modify already established connections. We contend that chronic stress in adulthood could disrupt the fine-tuned orchestra of axonal guidance cues and induce depressive-like behaviors. In the following section, and in Table 1, we list studies and review evidence suggesting that changes in guidance cue function induced by stress lead to depressive states.

**TABLE 1:** Preclinical and human studies linking guidance cues to chronic stress-induced PFC reorganization and MDD (findings from rodent and human studies do not necessarily match).

Guidance cue family	Evidence from rodent studies	References	Evidence from Post-mortem Human Studies	References
Netrin-1 receptors	↑ DCC expression in PFC of adult male mice susceptible to CSDS	Torres-Berrío et al., 2017	↑ DCC expression in mPFC of post-mortem brains from human male subjects with MDD who committed suicide	Manitt et al., 2013; Torres-Berrío et al., 2017
	DCC was one of the top predicted upstream regulators of transcriptomic changes in the PFC after exposed to unpredictable chronic mild stress in adult male mice	Musaelyan et al., 2020		
	Dysregulation of Netrin-1/DCC in mesolimbic DA regions of adolescent male mice susceptible to AcSD, but not in adults; differences in PFC DA connectivity seen between adults and adolescents subjected to AcSD	Vassilev et al., 2021		
Slit receptors	↓ SLIT1 in PFC of adult female mice exposed to chronic stress; PFC Slit1 Knockdown female mice showed increased depression-like behavioral abnormalities	Van der Zee et al., 2022	↓ SLIT1 is found in the adult PFC of women with depression compared to healthy controls, but not in men with MDD	Van der Zee et al., 2022
	↓ Robo3 gene expression in male mice exposed to CSDS, exhibiting depressive-like behaviors	Bondar et al., 2018	Methylation of Slit2 is associated with late-life MDD based on post-mortem PFC samples of elder populations	Hüls et al., 2020
Ephrin/Ephexin receptor	↑ EphA4 and ephexin1 in CSDS-induced susceptible adult male mice; upregulated EphA4 in PFC caused depression-like abnormalities in male mice	Zhang et al., 2017	↑ Phosphorylated EphA4 in the parietal cortex of depressed patients	Zhang et al., 2017
	↑ EphA2 receptors in PFC of female rats exposed to chronic unpredictable stress but not in males	Theriault et al., 2021	Ephrin receptors (*EPHA3, EPHA5*) genes were significantly associated with MDD in transcriptome studies from post-mortem PFC brain samples of MDD patients	Chang et al., 2014
	↓EphB2 in mPFC of male mice susceptible to chronic social defeat stress	Zhang et al., 2016	Gene networks with altered expression in the PFC of MDD patients were found to be involved in Ephrin signaling	Yuan et al., 2021
	↓EphB6 receptors in PFC of male mice exposed to CSDS may contribute to depression vulnerability	Guo et al., 2017	Differential methylation and expression for EphA2 found in PFC of patients who died by suicide	Romero-Pimentel et al., 2021
	Ephrin B signaling was found to be altered in PFC of mice undergoing chronic stress	Musaelyan et al., 2020		
	↓EphA10 expression in PFC of male mice exposed to CSDS	Bondar et al., 2018		
	Efnb2 KO in PFC of mice induces obvious fearless and reduced stress-induced behaviors	Sun et al., 2021		
	Ephrin signaling dysregulation in the PFC is associated with chronic stress in adult rats	Mychasiuk et al., 2016		
Semaphorin receptors	PlxnA1 KO mice ex hibited more stress-induced self-grooming, reduced prepulse inhibition, and decreased parvalbumin-expressing interneurons in mPFC of mice	Jahan et al., 2022	↑ Sema3F and neuropilin 1 were found as the differentially expressed genes in PFC of post-mortem brains from subjects with MDD	Tochigi et al., 2008; Goswami et al., 2013
	Sema3F knock-out male mice show reduced social interaction in SIT and other measures of depression-like behaviors	Matsuda et al., 2016	The *Sema3A* gene is associated with MDD in African American populations	Zhou et al., 2017

### The Netrin-1/DCC system

Netrin-1 is a secreted protein that attracts or repels axons and neurites by binding to transmembrane receptors, most notably deleted in colorectal cancer (*DCC)* and uncoordinated 5 (*UNC5)* homologues (Boyer and Gupton, 2018). Although peaking in neuronal cells during embryogenesis, netrin-1 and its receptors continue to be expressed in the adult matured brain, including in the PFC (Manitt et al., 2011; Goldman et al., 2013; Torres-Berrío et al., 2017). In adult mice, exposure to CSDS upregulates DCC receptor expression in PFC neurons, but only in those exhibiting a susceptible phenotype (Torres-Berrío et al., 2017). Experimentally increasing or decreasing DCC levels in PFC pyramidal neurons of adult male mice induces susceptibility or resilience, respectively, indicating a causal role. Altered expression of DCC in the PFC appears to be a persistent trait in MDD, supported by two independent post-mortem brain studies which show increased *Dcc* mRNA levels in the PFC of adult patients who died by suicide compared to non-psychiatric control subjects (Manitt et al., 2013; Torres-Berrío et al., 2017). A large and increasing number of studies show a tight link between genetic variation in the *Netrin-1/DCC* system and MDD. For a detailed description of the role of Netrin-1 and DCC receptors in stress-induced behavioral alterations in rodents and in MDD see Torres-Berrío et al. (2020a,b) and Vosberg et al. (2020).

Further confirmation of the Netrin-1/DCC system as a depression risk marker comes from a recent study that integrated multi-omics data. Li et al. (2020) conducted a genome-wide integrative analysis of depression and validated their findings in independent replications across different ethnic populations. The authors found that the *DCC* gene predicts the risk of depression in both Europeans and Han Chinese, with higher *Dcc* mRNA expression in the PFC associated with depression-relevant personality traits, cognitive function and putamen volumes in independent samples.

Entailing an additional layer of regulation, microRNA mechanisms mediate stress-induced changes in *DCC* receptor expression in the PFC of susceptible mice. Exposure to CSDS upregulates *Dcc* mRNA expression in the PFC by downregulating microRNA miR-218, a potent repressor of *DCC* receptors. In male mice, manipulating miR-218 in the PFC leads to corresponding changes in DCC levels in local neurons, inducing stress susceptibility or resilience, while in humans, miR-218 dysregulation is implicated in MDD (Manitt et al., 2013; Torres-Berrío et al., 2017). Due to the limitations in performing neurogenetic experiments on humans, the mechanism of how variations of DCC receptor expression in the PFC impact human behavior and psychopathology remains to be established. However, miR-218-mediated remodeling of dendritic spines in local neurons has been reported (Torres-Berrío et al., 2020b), in line with the strong influence of this pathway in the organization of dendritic structure and synaptic plasticity of the adult PFC (Goldman et al., 2013; Glasgow et al., 2020). Taken together, these findings suggest that alterations in the Netrin-1/DCC guidance cue system in the PFC predispose rodents to stress-induced susceptibility and depression-like behaviors, while also being associated with MDD in humans. For this reason, it constitutes a viable target for the development of new treatments for depression.

### The slit/robo system

Slits (*SLIT1-3*) are secreted proteins that act as a repulsive axon guidance cue, normally repelling growth cones by engaging roundabout (*ROBO*) class receptors (Gonda et al., 2020). Both netrins and Slits play an important role in midline axon guidance. Once a growing axon has crossed the midline in the developing brain, repulsion by Slits inhibits recrossing (Ypsilanti et al., 2010). Mutations in Slit-receptor *ROBO* induce midline guidance defects in humans (Battum et al., 2015). Recent studies have implicated Slits and their receptors in depression-related behaviors in mice, as well as in MDD in humans. For example, adult transgenic mice constitutively overexpressing human *Slit2* in whole-body exhibited increased depression-/anxiety-like behavior alterations assessed by sucrose preference test, open field test and elevated plus maze, with lower body weights compared to wild-type animals (Huang et al., 2021). Van der Zee et al. (2022) revealed a *SLIT1* downregulation in the ventromedial PFC of women with MDD in comparison with healthy controls, an effect not seen in men with depression. Similarly, a sex-specific pattern of *Slit1* downregulation was discovered in the ventromedial PFC of female mice exposed to chronic variable stress but not in males. After performing a knockdown of *Slit1* expression in the ventromedial PFC of both sexes of mice, female mice had a sex-specific elevation in depression-like behaviors, as well as a decreased dendritic arborization and excitability of ventromedial PFC pyramidal neurons, with both findings absent in males. For a more extensive list of studies implicating Slits in stress-induced depression-like behaviors and MDD, please consult Table 1.

### The ephrin, ephexin/eph receptor system

Unlike other conventional guidance cues, which can be either membrane bound or secreted, all ephrins are membrane-bound ligands that activate Eph receptors (Ephs) on the surface of neighboring cells, to induce either attraction or repulsion (Egea and Klein, 2007). In some cases, ephrin- and Eph-expressing cells can engage in “reverse signaling” in which ephrin transduces a signal in its expressing cell while the Eph receptor acts as the ligand (Holland et al., 1996; Lisabeth et al., 2013). Both “forward” and “reverse,” termed “bidirectional signaling,” are important for the proper formation and maintenance of local circuit microarchitecture (Murai and Pasquale, 2003; Lisabeth et al., 2013).

Zhang et al. (2017) investigated the role of the ephrin receptor *EphA4*– ephexin1 signaling in depressive-like behaviors and examined if systemic administration of rhynchophylline, an EPHA4 inhibitor, has an antidepressant-like effect. Adult male mice were subjected to CSDS, and afterward susceptible mice showed increased levels of phosphorylated EPHA4 and ephexin1 in the PFC and hippocampus. Overexpression of EPHA4 in the PFC induced depressive-like phenotypes and rhynchophylline reversed the phenotypes in these mice. Furthermore, Zhang et al. (2017) corroborates the importance of these findings by showing that individuals with MDD also have a higher level of phosphorylated EPHA4 in the parietal cortex, compared with healthy controls. Chang et al. (2014) conducted meta-clustering of gene expression links in 11 transcriptome studies from post-mortem PFC brain samples of MDD and non-psychiatric control subjects. Further implicating the ephrin pathway in depression, they found that Ephrin receptors (EPHA3, EPHA5) genes were among 88 genes that were significantly associated with MDD by GWAS, and with medical illnesses with increased clinical risk of depression, but not for other illness. These findings suggest that ephrin and ephexin signaling through Eph receptors in the PFC is a potential marker of depressive-like phenotypes and is associated with MDD, making this pathway a potential therapeutic target for depression. For additional studies implicating the ephrin, ephexin/Eph receptor pathway to stress and depressive-like behavior or MDD, please consult Table 1.

### The semaphorin/plexin, neuropilin system

Semaphorins represent a family of thirty proteins, grouped into eight classes, seven of which are found in animals (*SEMA1-7*) and one in viruses (*SEMAV*). Semaphorins can either be secreted, membrane spanning, or membrane associated and are primarily axonal repellents, activating complexes of cell-surface receptors called Plexin family proteins (Plexin A1-4) and Neuropilin family proteins (Neuropilin-1 and -2) (Kolodkin et al., 1993). For a comprehensive review of semaphorin function in the adult brain, please consult Alto and Terman (2016), Carulli et al. (2021).

SEMA3F, a secreted semaphorin, has been implicated in depressive-like behaviors in male mice. Matsuda et al. (2016) showed that SEMA3F knockout male mice show a reduced score in the social interaction test compared with controls, among multiple other neurological findings. In human psychopathology research, a GWAS study identified a risk variant in the *SEMA3A* gene associated with comorbid alcohol dependence and depression in African American participants (Zhou et al., 2017). For additional studies implicating chronic stress or MDD to changes in semaphorin signaling, please consult Table 1.

### Morphogens

Recent studies highlight the roles of *WNT* and *SHH* signaling in depression and depression-like behaviors, possibly *via* BDNF-mediated processes (Voleti and Duman, 2012; Tayyab et al., 2018). *WNT* signaling may regulate depression-like behaviors by altering adult neurogenesis, dendritic morphology, synaptic plasticity and synaptic transmission. For a detailed review on the role of *WNT* signaling, please refer to Voleti and Duman (2012). The role of morphogens on stress-induced alterations in the adult PFC has not been sufficient investigated.

### Cell adhesion molecules

Several non-conventional guidance molecules such as laminin, tenascins, proteoglycans, N-CAM, and L1-CAM participate in axonal guidance. If considered together, a large body of evidence implicate these molecules in depression-like behaviors. For instance, the polysialylated (PSA) form of N-CAM is involved in the development and migration of neurons in the immature vertebrate brain (Quartu et al., 2008), while also participating in neurite and synaptic remodeling (Varea et al., 2007). Although expressed throughout the lifetime, it is required for synaptogenesis and structural plasticity in adulthood, with multiple studies implicating it in the structure and function of the adult male rat PFC (Varea et al., 2005; Castillo-Gómez et al., 2008, 2011; Rutishauser, 2008). PSA-NCAM mediates synaptic plasticity, neurogenesis, signaling by neurotrophic factors and inflammatory messengers in the brain, all relevant processes in depression-like behaviors (Saini et al., 2020). This makes PSA-NCAM a viable candidate for increasing neuroplasticity, a hallmark in the modern hypothesis of treatment for MDD. Chronic treatment with the tricyclic antidepressant imipramine increased the expression of polysialylated N-CAM in the PFC and hippocampus (Sairanen et al., 2007). Similarly, chronic fluoxetine treatment increased the PSA N-CAM expression in the PFC (Varea et al., 2007). However, selective cleaving of polysialylation moieties using endoneuraminidase N inhibited the antidepressant efficacy of fluoxetine in a chronic unpredictable stress model for depression-like behaviors, likely by disrupting the interaction of PSA-NCAM with the D2 dopamine receptor in the medial PFC (Castillo-Gómez et al., 2008, 2011; Wainwright et al., 2016). Due to the structural nature of PSA-NCAM, direct administration using purified protein would be an impediment to its utility as an antidepressant, which led researchers to use peptide mimetics of NCAM to circumvent this barrier (Hansen et al., 2009; Køhler et al., 2010; Xu et al., 2014). Turner et al. (2019) compared three of these peptide mimetics in rats and found that each one had either anxiolytic or antidepressant effects with their own intrinsic kinetics, which consolidates this unconventional area of depression research. These findings suggest that chronic stress downregulates the expression of the PSA-NCAM in the PFC, and that PSA-NCAM-mediated synaptic plasticity is necessary for antidepressant action. For additional work linking cell adhesion molecules to MDD or depression-like behaviors, please read the work of Laifenfeld et al. (2005a,b).

## Beyond the PFC: Hippocampus

The hippocampus is another brain area in which disruption in axonal guidance signaling can lead to depression-like phenotypes. Notably, neogenin, a multifunctional transmembrane receptor, participates in adult hippocampal neurogenesis. Loss of neogenin reduced dendritic branches and spines, impaired glutamatergic neurotransmission, and mice with depletion of neogenin in adult neural stem cells or neural progenitor cells showed depressive-like behavior (Sun et al., 2018). Gui et al. (2021) compared the hippocampal transcriptional features between four models [i.e., chronic unpredictable mild stress (CUMS), CSDS, learned helplessness and MDD patients]. The authors found that axonal guidance signaling was one of the seven significantly enriched pathways in all four models (Gui et al., 2021). Li et al. (2022) reported that EPHA4 expression is increased in the excitatory neurons in the hippocampus of mice subjected to CUMS, and knockdown of *EphA4* prevented depression-like behaviors. EPHA4 levels were significantly higher in MDD samples compared to control individuals (Li et al., 2022). The administration of fluoxetine for 4 weeks restored dysregulated EPHA4 levels in fluoxetine responder rats compared to antidepressant resistant rats (Li et al., 2014). This suggests that ephrin signaling in the hippocampus is implicated in the antidepressant effect.

Semaphorin 3B in the hippocampus may play a role in inducing depression-like behaviors. Levels of SEMA3B protein are decreased in the hippocampus and serum of chronic mild stress (CMS)-treated mice (Du et al., 2022). Increasing the levels of *SEMA3B* in the hippocampus or the lateral ventricles, improved CMS-induced depression-like behaviors and increased resilience to acute stress by increasing dendritic spine density in hippocampal neurons (Du et al., 2022).

Emerging findings suggest that *Shh* is involved in adult hippocampal neurogenesis and may have an antidepressant effect. Yao et al. (2016) investigated the role of *Shh* in electroconvulsive (ECT) therapy in rats. ECT is often used in treatment-resistant depression and severe cases of depression. ECT induced the proliferation of hippocampal neural progenitor cells in rats, and blockade of Shh signaling with cyclopamine completely inhibited ECT-induced neural progenitor cell proliferation (Yao et al., 2016). However, it is yet to be investigated how *Shh* signaling mechanisms induce depression-like behaviors.

## Biomarkers for precision medicine

Measuring subtle changes in axonal guidance cue signaling in the PFC could offer a novel approach to precision medicine and the treatment of depression. MicroRNAs are promising molecules that fit this description, as their expression level has previously been well correlated with structural and functional changes in the PFC, as exemplified by mir-218 in a previous section, and they can readily be detected in peripheral fluids. It is conceivable that changes in these biomarkers could correlate between the brain and peripheral fluids under normal circumstances and could also be used as a readout of treatment efficacy. Chronic stress-induced changes in the PFC in rodents, often in axonal guidance signaling pathways, may coincide with changes in levels of biomarkers (other than mir-218) in peripheral fluids, such as saliva, and peripheral blood (Torres-Berrío et al., 2020b; Morgunova and Flores, 2021). For example, Fan et al. (2014) reported that compared to age and gender-matched control subjects, in depressed patients, 25 of 26 identified miRNAs were upregulated. These miRNAs are all involved in pathways related to axon guidance, WNT signaling, ERBB signaling, mTOR signaling, VEGF signaling, and long-term potentiation (Lopizzo et al., 2019).

## Conclusion

Depression is a chronic disabling disease, often induced by chronic stress. It is paramount to unravel the cellular and molecular pathophysiology of depression that could guide strategies to prevent its onset and help accelerate the development of novel therapeutics. In this review, we gathered accumulating evidence of the roles of axonal guidance cues in depression-like behaviors in rodents. We also discussed supporting data from human studies emphasizing the importance of further elucidating the involvement of guidance cues in MDD. We highlight the need of conducting translational research connecting human and rodent observations, that can help us better determine how and when alteration and disruption in the signaling pathways of various axonal guidance cues can affect vulnerability. Depression is twice more likely in women than men, and there is a great need to understand how and why this sex-difference arises. Since most preclinical studies have been conducted primarily on male rodents, relatively little is known about how biological sex, together with different behavioral coping strategies, induce male and female vulnerability. To move forward, rodent models should always include females or carefully justify the use of only one sex. If existing models do not work in females, new models need to be devised.

## Author contributions

AM, RGA, and CF wrote the manuscript. All authors contributed to the article and approved the submitted version.
